# A novel, free regional conference model to increase interest and engagement of medical trainees in pediatrics and pediatric gastroenterology research

**DOI:** 10.1002/jpr3.70252

**Published:** 2026-10-12

**Authors:** Mahnoor Memon, Shahzaib Khan, Christina Guillen, Thomas Wallach

**Affiliations:** ^1^ College of Medicine SUNY Downstate Health Sciences University Brooklyn New York USA; ^2^ Department of Pediatrics, Division of Pediatric Gastroenterology SUNY Downstate Health Sciences University Brooklyn New York USA; ^3^ Department of Pediatrics SUNY Downstate Health Sciences University Brooklyn New York USA

**Keywords:** health equity, medical education, pediatric gastroenterology workforce, physician‐scientist pipeline, trainee engagement

## Abstract

Declining interest in pediatrics, contraction of the pediatric gastroenterology fellowship applicant pool, and the long‐standing erosion of the physician–scientist workforce threaten the future of pediatric academic medicine. Cost and logistical barriers to traditional academic conferences disproportionately affect trainees from underrepresented or resource‐limited backgrounds and may compound these pipeline pressures. We developed Eats, Streets, and Beets (ESB), a free, trainee‐centered regional pediatric nutrition conference held in Brooklyn, NY, combining faculty‐led symposia, guaranteed podium presentations for every accepted abstract, and a community block party. A post‐conference survey (*n* = 12; response rate 63.2%) demonstrated high overall satisfaction (mean 8.25/10) and self‐reported increases in interest in research, pediatrics, and pediatric gastroenterology and nutrition. Trainee‐only programming, podium‐only presentations, and community engagement were most strongly associated with increased interest. This low‐cost, replicable model may help reinvigorate trainee engagement across the pediatric and pediatric gastroenterology pipelines.

## Abstract (highlights)

**What is Known:**

- The U.S. physician–scientist workforce has steadily contracted, with fewer trainees entering and remaining in research careers.
- Cost, travel, and time‐away requirements of traditional academic conferences disproportionately limit participation by trainees from underrepresented or resource‐limited backgrounds.
- Pediatrics and pediatric subspecialty fellowships, including pediatric gastroenterology, have experienced declining match fill rates and applicant volumes in recent cycles, as well as declining interest in research.

**What is New:**

- A free, regionally focused, trainee‐centered conference with universal podium presentations and embedded community engagement is feasible and well received.
- Participation was associated with self‐reported gains in interest in research, pediatrics, and pediatric gastroenterology and nutrition, with the strongest associations for trainee‐only, podium‐only, and community‐engaged programming elements.
- This model offers a low‐cost, scalable strategy for academic medical centers to counter declining interest in pediatrics and pediatric subspecialty research careers.

## 1 INTRODUCTION

The physician–scientist workforce continues to face long‐term pipeline challenges, including declining participation and persistent inequities in recruitment and retention.^1^ These pressures are particularly acute in pediatrics: in the 2024 and 2025 Main Residency Match, categorical pediatrics fill rates fell substantially relative to historical norms, with the 2024 cycle leaving 252 categorical pediatric positions unfilled and the 2025 cycle showing continued weakness in pediatric matching.^2^ Pediatric subspecialty fellowships have shown parallel softening; in the most recent NRMP Pediatric Specialties Match, 21.7% of positions across pediatric subspecialties went unfilled, and pediatric gastroenterology specifically has experienced reductions in applicant‐to‐position ratios in recent cycles.^3^ These inequalities are reinforced by structural barriers that disproportionately affect trainees who do not have traditional academic access at well‐resourced institutions. Prior work has highlighted that lack of “incidental access” to research and limited curricular visibility of scholarly work are associated with a lower likelihood of pursuing research‐oriented careers among medical trainees.^4^ Participation in professional conferences and academic development activities often requires payment of registration fees, travel costs, lodging, and protected time. Total expenses for a single conference typically exceed $1500 per event^5^ In addition, residents and fellows must navigate scheduling constraints related to clinical duties, call schedules, examinations, and institutional leave policies, which may result in loss of clinical time or income and reduced feasibility of attendance.^6^ These financial and logistical barriers disproportionately limit participation among trainees from underrepresented or resource‐limited backgrounds.^7^ Diverse research teams have been shown to generate more innovative solutions, higher‐impact scientific output, and improved problem‐solving compared with homogeneous groups, providing an empirical rationale for efforts to broaden participation in academic medicine and research training.^8^ Programs designed to reduce such barriers may represent actionable strategies to strengthen and diversify the pediatric and pediatric gastroenterology physician–scientist pipeline.

To address these barriers, we developed *Eats, Streets, and Beets* (ESB), a trainee‐focused pediatric nutrition conference designed to reduce access barriers through free, trainee‐centered programming with embedded faculty mentorship and structured community engagement. The conference was held at SUNY Downstate Health Sciences University in Brooklyn, New York. The location and accessibility allowed participants to attend via public transportation, minimizing travel expense and time away from clinical duties and home. The program opened with nutrition‐focused faculty symposium talks on practically oriented topics, including dietary contributions to dermatologic health, behavioral approaches to selective eating in children, and prevention of food allergy. These sessions were followed by abstract presentations, each delivered as a podium presentation at no cost to the trainee. All participants were automatically considered for the Golden Beets Award, which recognizes a trainee demonstrating mastery of content and confidence in presentation, emphasizing trainee performance independent of mentor seniority or funding. Participants included medical students, residents, and fellows from institutions across the New York metropolitan area, and faculty from three institutions serving as introductory speakers and as judges for trainee presentations. The community block party, including children's play and craft areas, book giveaways, locally sourced food, a DJ, and performances by Afro‐Caribbean dance troupes and bands, provided focal clinical advice and access to supporting institutions for our community, and contextualized trainee and faculty scholarly work within the community served by our institution.

We hypothesized that this approach would generate trainee‐reported interest in research, pediatrics, and pediatric gastroenterology and nutrition, and we assessed this hypothesis using a post‐conference survey.

## 2 METHODS

### 2.1 Ethics statement

The study was reviewed by the SUNY Downstate Health Sciences University Institutional Review Board and deemed exempt from full review (#2379354), as it involved anonymous surveys with minimal risk to participants. Informed consent was implied by voluntary completion of the survey; no identifying information was collected.

### 2.2 Conference and survey design

Following the conference, participants completed a post‐conference survey assessing training level, prior conference participation, overall experience, and perceived impact of participation on interest in research, pediatrics, and pediatric gastroenterology and nutrition using a 1–10 Likert‐type scale (Figure S1). The conference and data collection were conducted in September 2025.

### 2.3 Statistical analysis

Descriptive results were summarized as frequencies, percentages, and means. Associations between conference features and trainee‐reported outcomes were assessed using Spearman's rank‐order correlation (*ρ*). A two‐sided *p* < 0.05 was considered statistically significant. Analyses were performed using standard statistical software.

## 3 RESULTS

In total, 27 trainees attended ESB, including 19 abstract presenters; an additional nine faculty members and three invited faculty speakers were present (Table 1). Of the 19 trainee presenters, 12 responded to the survey (response rate 63.2%). Participants represented multiple trainee levels (Figure 1A). Eighty‐three percent reported interest in a research‐oriented career and 75% in pediatrics. Overall satisfaction with the ESB conference was high (mean 8.25/10), and participants reported positive impacts on interest in pediatrics (mean 8.0/10), interest in research (8.58/10), and interest in pediatric gastroenterology and nutrition (8.67/10) (Figure 1B). Unique features of the conference scored highly, with positive ratings for the universal use of podium presentations (8.92/10), performances (8.75/10), community party (8.67/10), trainee‐only nature (8.44/10), and free admission (8.27/10) (Figure 1C).

**Table 1:** Survey participants and demographics.

Characteristic	*N* = 12
Gender	
Female	8 (66.7)
Male	4 (33.3)
Race	
Caucasian	5 (41.7)
Black/African American	3 (25.0)
Asian	4 (33.3)
Ethnicity	
Non‐Hispanic	9 (75.0)
Hispanic	3 (25.0)
Training level	
Medical student	6 (50.0)
Resident	4 (33.3)
Fellow	2 (16.7)
Non‐MD advanced degree	1 (8.3)
Interest in pediatrics	9 (75.0)
Interest in research	10 (83.3)
Conferences attended	
Mean (SD)	2.4 (1.7)
Range	0−5

*Note*: Values are presented as *n* (%) unless otherwise specified.

Abbreviation: SD, standard deviation.

**Figure 1:**
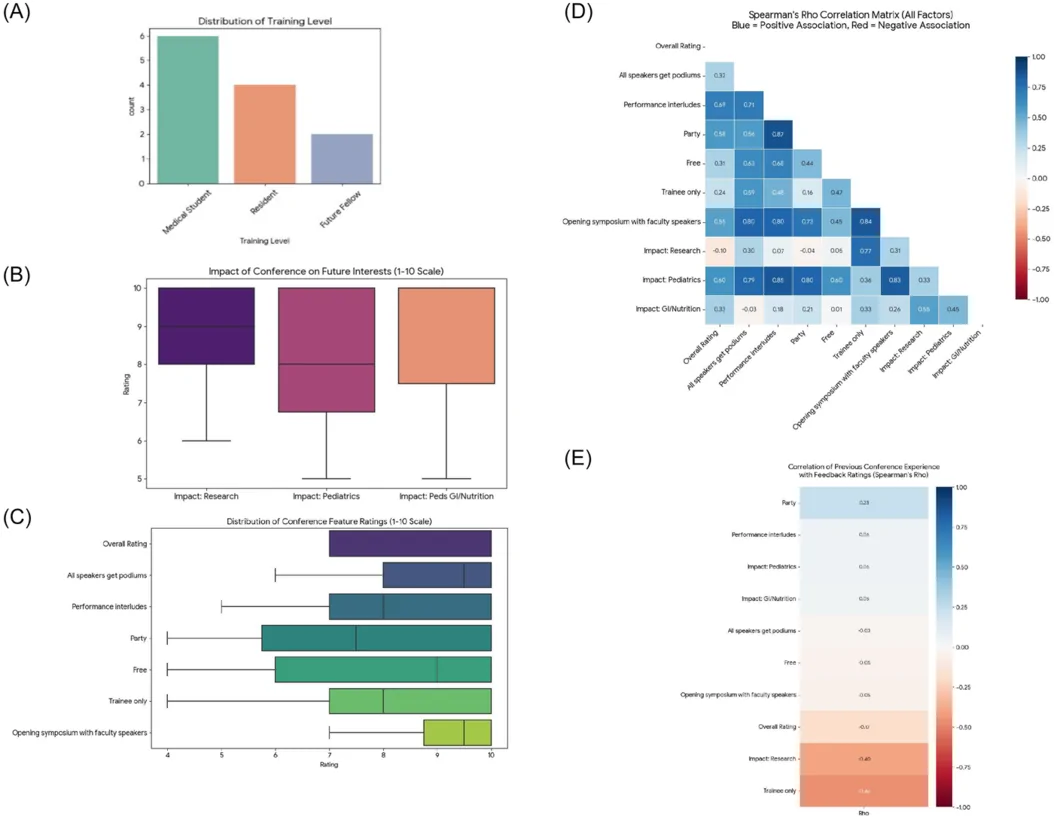
Conference feedback analysis. (A) Distribution of participant training levels (*N* = 12). (B) Box plots showing impact of conference on future interests in research, pediatrics, and pediatric gastroenterology/nutrition (scale 1–10). (C) Stacked horizontal bars showing distribution of ratings for seven conference features. (D) Spearman's rank correlation matrix showing relationships between all measured variables; blue indicates positive associations, red indicates negative associations. (E) Correlation analysis of previous conference attendance with feedback ratings across multiple dimensions. All correlation values significant at *p* < 0.05 are shown in bold.

Several patterns emerged (Figure 1D). The strongest association with increased interest in research was the trainee‐only nature of the conference (*ρ* = 0.77, *p* = 0.0145). The factors most strongly associated with increased interest in pediatrics were the podium‐only structure (*ρ* = 0.79, *p* = 0.0024), the community performances (*ρ* = 0.85, *p* = 0.0005), and the community party (*ρ* = 0.80, *p* = 0.0018). The opening end‐user–focused symposium was also well received, with positive correlations with increased interest in pediatrics (*ρ* = 0.83, *p* = 0.0008), favorable view of the trainee‐only conference structure (*ρ* = 0.84, *p* = 0.0041), and favorable view of the community party (*ρ* = 0.73, *p* = 0.0073). When assessed against prior conference experience, no significant associations emerged; however, exploratory trends suggested that the perceived value of trainee‐only programming and impact on future research goals were concentrated among less experienced participants, whereas the perceived value of the community party concentrated in participants with less prior conference exposure (Figure 1E).

## 4 DISCUSSION

The ESB conference was designed to reduce barriers to trainee participation while maximizing opportunities for podium presentation, faculty mentorship, and community engagement. Our post‐participation survey suggests that these objectives were met. Integration of a free, trainee‐centered, community‐engaged event was associated with self‐reported increases in interest in research, pediatrics, and pediatric gastroenterology and nutrition. These findings should be interpreted in light of important limitations, including the absence of pre‐intervention data, a small sample size, and probable selection bias given participants’ high baseline interest in research. Even so, the model addresses several converging workforce pressures in pediatric academic medicine that warrant explicit discussion.

Interest in pediatrics as a specialty has measurably declined in the most recent Main Residency Match cycles. The 2024 NRMP cycle saw the pediatrics fill rate drop from 97.1% to 91.8%, with 252 categorical pediatric positions unfilled—an outcome without recent historical precedent—and the 2025 cycle showed only modest recovery despite an increase in offered positions.^2^ This contraction has triggered formal responses from the American Academy of Pediatrics and the Association of Pediatric Program Directors, who have called for structural changes in how pediatrics is presented to medical students and how trainees are recruited and retained.^9^ Within this context, low‐barrier, high‐engagement programming that places trainees at the center of the academic experience—as in the ESB model—may help re‐establish pediatrics as a visible, vibrant, and intellectually compelling career path for medical students and early residents.

These pressures are amplified at the subspecialty level. In the 2025 NRMP Pediatric Specialties Match, 21.7% of pediatric subspecialty positions went unfilled overall, and pediatric gastroenterology—while still relatively well filled—has experienced a contraction in applicant‐to‐position ratios and a narrowing of the pipeline from which programs can recruit.^3^ Sustained underfilling threatens access to subspecialty care for children, particularly in safety‐net and resource‐limited settings. Embedding pediatric gastroenterology and nutrition content within a regionally accessible, trainee‐facing forum, as we did with ESB, offers an early and concrete touchpoint with the field for medical students and pediatric residents who might not otherwise consider subspecialty training. Notably, the strongest correlations in our data linked the podium‐only and community‐engaged elements of the conference to increased interest in pediatric gastroenterology and nutrition, suggesting that visibility, ownership of scholarly work, and a sense of belonging may be more important recruitment levers than purely didactic exposure.

Finally, ESB sits within the broader, decades‐long contraction of the physician–scientist workforce. The proportion of U.S. physicians engaged in research has fallen from approximately 4.75% in the 1980s to roughly 1.5% today, and successive NIH Physician–Scientist Workforce Working Group reports have documented declining inflow and accelerated outflow from research careers.^1^, ^10^ Pediatrics has been disproportionately affected, given longer training pathways, lower compensation relative to adult subspecialties, and fewer protected research opportunities during fellowship. Interventions that lower the cost of entry to scholarly activity, normalize podium presentation early in training, and connect research to community impact may help counter these trends. ESB is not a substitute for sustained, longitudinal mentorship or protected research time, but it provides an accessible on‐ramp—particularly for trainees whose institutions lack the resources to send them to national meetings.

Operationally, ESB achieved its aims through geographically accessible, no‐cost programming at a centrally located urban site in Brooklyn, allowing participants to attend without airfare, lodging, or extended absences from clinical duties. Elimination of registration fees and travel costs lowered barriers for trainees from resource‐limited backgrounds. Universal podium presentation provided equitable opportunities for all accepted abstracts and promoted active dialogue across research interests, supporting authentic participation in scholarly exchange.

This approach has some limitations, relying as it does on density of both medical trainees and urban density and transit to maintain extraordinarily low cost of participation. However, multiple major metro areas fit this need ideally, including Washington/Baltimore, Boston/Providence, Atlanta, Los Angeles, Dallas, St. Louis, San Francisco, Chicago, and more. While the model may not be universally applicable in low‐density regions, similar approaches aimed at lowering participation costs may be effective as the bulk of trainees will have access to vehicles. By focusing on regional iterations, this approach could be scaled in concert to support engagement in pediatrics and research nationwide, and even areas with poor density will be able to offer more accessible programming.

Taken together, our findings suggest that a free, culturally relevant, and trainee‐centered conference can produce meaningful improvements in self‐reported engagement and interest across pediatrics, pediatric gastroenterology and nutrition, and research. The model is replicable at scale in major metropolitan areas and can function as infrastructure for regional mentorship networks, while embedding community‐engagement activities that link academic work to local public health needs. By broadening participation across socioeconomic, geographic, and institutional backgrounds, ESB expanded the range of voices contributing to scientific dialogue, which has previously been shown to improve research productivity and rigor.^8^

## 5 CONCLUSION

ESB demonstrates that trainee‐focused, accessible regional programming may help reduce disparities in academic medicine by decentralizing opportunities for scholarly engagement. Implementation of similar low‐cost, community‐embedded models may serve as a meaningful adjunct to broader efforts to address declining interest in pediatrics, the contracting pediatric gastroenterology applicant pool, and the long‐standing erosion of the physician–scientist workforce.

## CONFLICTS OF INTEREST STATEMENT

The authors declare no conflicts of interest.

## Supporting information

Supplemental Figure 1 Survey Instrument.

## Data Availability

All data are presented within this manuscript; raw data are available upon reasonable request from the corresponding author.
