# Supplementary material for: A novel, free regional conference model to increase interest and engagement of medical trainees in pediatrics and pediatric gastroenterology research
Source: JPGN Rep. 2026 Oct 12:10.1002/jpr3.70252. Online ahead of print. doi: 10.1002/jpr3.70252 (PMC13656292; doi:10.1002/jpr3.70252)
Supplement: Supplementary file 1 [file JPR3-9999-0-s001.pdf]

ESB Feedback

Thank you for participating in our first Eats, Streets, Beats Nutrition Conference! We would appreciate your feedback and participation in our educational research as to how the event was received and how we can better support you as professionals in training.

PARTICIPANT BACKGROUND

1.

What is your current level of training?

- ■ Medical Student
- ■ PhD
- ■ Resident
- ■ Other: _______________

Mark only one oval.

2.

How many conferences have you attended?

____

3.

Do you have an interest in a research-oriented career?

- ■ Yes
- ■ No

Mark only one oval.

4.

Do you have an interest in pediatrics?

- ■ Yes
- ■ No

Mark only one oval.

5.

Do you have additional degrees?

- ■ Yes
- ■ No

Mark only one oval.

6.

What other degrees do you have?

- ■ PhD Science
- ■ PhD Humanities
- ■ MA Science
- ■ MA Humanities
- ■ MPH
- ■ Other: _______________

Check all that apply.

CONFERENCE RATING

Mark only one oval per row. (1 = Very poor / Not important; 10 = Excellent / Very important)

	1	2	3	4	5	6	7	8	9	10
**7.** On a scale of 1–10, how would you rate this conference overall?	■	■	■	■	■	■	■	■	■	■

IMPORTANCE OF CONFERENCE FEATURES

On a scale of 1–10, how important was your experience with the following aspects of the conference?

	1	2	3	4	5	6	7	8	9	10
**8.** All speakers get podiums	■	■	■	■	■	■	■	■	■	■
**9.** Performance interludes	■	■	■	■	■	■	■	■	■	■
**10.** Party	■	■	■	■	■	■	■	■	■	■
**11.** Free	■	■	■	■	■	■	■	■	■	■
**12.** Trainee only	■	■	■	■	■	■	■	■	■	■
**13.** Opening symposium with faculty speakers	■	■	■	■	■	■	■	■	■	■

IMPACT ON FUTURE INTERESTS

On a scale of 1–10, with 10 being substantially more and 1 being substantially less, give your rating for the following:

	1	2	3	4	5	6	7	8	9	10
**14.** How did your participation impact your interest in research?	■	■	■	■	■	■	■	■	■	■
**15.** How did your participation impact your interest in pediatrics?	■	■	■	■	■	■	■	■	■	■
**16.** How did your participation impact your interest in pediatric gastroenterology or nutrition?	■	■	■	■	■	■	■	■	■	■

Supplemental Figure 1. ESB Conference Feedback Survey Instrument.
